# Review on the Role of Salivary Biomarkers in the Diagnosis of Mild Traumatic Brain Injury and Post-Concussion Syndrome

**DOI:** 10.3390/diagnostics13081367

**Published:** 2023-04-07

**Authors:** Ioannis Mavroudis, Foivos Petridis, Ioana-Miruna Balmus, Alin Ciobica, Dragos Lucian Gorgan, Alina Costina Luca

**Affiliations:** 1 Department of Neurology, Leeds Teaching Hospitals NHS Trust, Leeds LS9 7TF, UK; 2 Faculty of Medicine, Leeds University, Leeds LS2 9JT, UK; 3 Third Department of Neurology, Aristotle University of Thessaloniki, 541 24 Thessaloniki, Greece; 4 Department of Exact Sciences and Natural Sciences, Institute of Interdisciplinary Research, Alexandru Ioan Cuza University of Iasi, Alexandru Lapusneanu Street, No. 26, 700057 Iasi, Romania; 5 Department of Biology, Faculty of Biology, Alexandru Ioan Cuza University of Iasi, B dul Carol I, No. 11, 700506 Iasi, Romania; 6 Department of Mother and Child, Medicine—Pediatrics, “Grigore T. Popa” University of Medicine and Pharmacy, 16, Universitatii Street, 700115 Iasi, Romania

**Keywords:** mild traumatic brain injuries, post-concussion syndrome, diagnosis, prognosis, outcomes, saliva, S100B, neurofilament light chain, microRNA, extracellular vesicles, exosomes

## Abstract

(1) Background: While mild traumatic brain injuries (TBIs) are a major public health issue, post-concussion syndrome (PCS) remains a controversial entity. In both cases, the clinical diagnosis is mainly based on the symptoms and brain imaging evaluation. The current molecular biomarkers were described from blood and cerebrospinal fluid (CSF), yet both fluid collection methods are invasive. Saliva could be preferred in molecular diagnosis due to its non-invasive and non-expensive methods of acquisition, transport, and samples processing. (2) Objectives: In the present study, we aimed to review the latest developments in salivary biomarkers and their potential role in diagnosing mild TBIs, and PCS. (3) Results: In TBIs and PCS, a few novel studies focusing on salivary biomarkers have emphasized their importance in diagnosis. The previous studies mainly focused on micro RNAs, and only a few on extracellular vesicles, neurofilament light chain, and S100B. (4) Conclusions: The combination between salivary biomarkers, clinical history and examination, self-reported symptoms, and cognitive/balance testing can provide a non-invasive alternative diagnostic methodology, as compared to the currently approved plasma and cerebrospinal fluid biomarkers.

## 1. Introduction

Head injuries, mild TBIs in particular, are a significant concern due to their potential to create long-term health consequences, such as post-concussion syndrome (PCS) and chronic traumatic encephalopathy (CTE) [1].

Despite the fact that the underlying mechanism of concussion is still not fully described, it has been shown that the stretching and disruption of neuronal and axonal cell membranes actively participate as triggers of neurometabolic cascade activation, leading to neuronal and axonal injury and death. On the other hand, these mechanical damages to brain tissues could determine neuroinflammation and microglia activation that could further contribute to the short and long-term complications [2,3].

Several classification systems for TBIs have been proposed to reflect the pathophysiological aspects. However, since most of these classification systems and diagnostic criteria are based on clinical observations and symptomology, there are only a few that are widely used in diagnosis [4]. In this way, TBIs can be classified based on severity, pathoanatomic type, outcome, and prognosis [5]. The Glasgow Coma Scale (GCS) is commonly used to classify TBIs as mild (GCS score of 13–15), moderate (GCS score of 9–12), or severe (GCS score of 3–8) [6]. The extent of post- or peri-traumatic amnesia is another important factor in determining TBI severity. A TBI with post-traumatic amnesia of 1–24 h is considered moderately severe, but more recent classifications of moderate TBI require post-traumatic amnesia lasting beyond 24 h [7,8].

One widely accepted TBI classification system is the Mayo System (Table 1), which categorizes TBI as possible, probable—mild, and definite moderate-severe [8]. However, the most problematic aspect of this classification system remains the mild TBI, as their criteria refer to blurred vision, confusion, headache, or nausea, any loss of consciousness for less than 30 min, post-traumatic amnesia for less than 24 h, and a depressed, basilar, or linear skull fracture with intact dura matter that can often be missed during the initial imaging scans. Moreover, GCS could be administered to the patients at 30 min following the head trauma due to their loss of consciousness. The Mayo Classification System also requires the exclusion of other causes of impaired consciousness. Furthermore, the additional evidence of brain hematoma, hemorrhage, contusions, or ruptured dura matter categorizes the observed TBI as moderate-severe [9], even though mild TBIs could also be characterized by significant changes of the brain molecular pathways.

## 2. Post-Concussion Syndrome—Epidemiology and Diagnosis Criteria

PCS is a sequela of mild TBI, with a prevalence rate of 29–90% among patients who have suffered a head injury [1]. There is no universally accepted definition for PCS, but it is typically characterized by at least three symptoms, such as headache, fatigue, irritability, dizziness, balance issues, disturbed sleep, poor memory and concentration, and increased sensitivity to light and noise. These symptoms appear shortly after a head injury and can persist for weeks or months. When the symptoms persist for more than six months or one year, the condition is referred to as prolonged PCS (PPCS) [10].

The more benign International Classification of Diseases, Tenth Revision (ICD-10) diagnostic criteria for PCS include a history of TBI and three or more symptoms, such as headache, dizziness, fatigue, irritability, insomnia, concentration or memory disturbance, and intolerance to stress, alcohol, and emotions, providing a psychogenic approach in diagnosis [1,11,12]. On the other hand, the American Psychiatric Association’s Statistical Manual of Mental Disorders, Fifth Edition (DSM-5) defines PCS as a major or mild neurocognitive disorder due to traumatic brain injury, which requires evidence of traumatic brain injury with any of the following symptoms: loss of consciousness, post-traumatic amnesia, disorientation and confusion, new onset of seizures, anosmia, or hemiparesis; this approach focuses more on the post-TBI cognitive decline evaluation, thus offering a neurogenic approach in diagnosis and recovery prognosis [12,13].

Recent studies described the consequences of post-concussive injury as being persistent for a longer period, leading to the notion that the long-term effects of PCS go beyond just neurological traits. In this context, Clark et al. recently suggested that the concept of PCS could not be unidimensional but framed in a bio-psycho-socio-ecological model, in a facile manner [14]. The neurocognitive symptoms, for example, occur directly after the TBI or immediately after regaining consciousness, and could persist for longer than the acute post-injury period [13]. Moreover, it was shown that the neurocognitive impairments seen in PCS result due to complex mechanisms involving neurodegeneration and neuroinflammation, the latter starting from the acute post-injury period.

Thus, not only the psychogenic and neurogenic approach in diagnosis could be considered, but also the molecular approach. While multiple molecular biomarkers have been recently described in mild TBIs and PCS, their specificity is still under debate. Moreover, as non-invasive evaluation is preferred in emergency medicine, recent research has shown that salivary biomarkers can be essential in diagnosing PCS. As saliva contains a wide range of biomolecules that are indicative of various physiological processes, such as hormones, proteins, and microRNAs, several salivary molecules, including S100B, neurofilament light chain (NfL), micro RNAs, and exosome vesicle proteins, have been found to be associated with mild TBIs and PCS; these are thus potent specific biomarkers, offering a promising tool for the early detection and management of PCS and CTE. In the present study, we aimed to review the latest developments in salivary biomarkers and their potential role in diagnosing mild TBI, and PCS.

## 3. Salivary Biomarkers

Considering the vital physiological processes that commonly occur within the oral cavity and its main implication in digestion and hygiene, saliva often consists of various transitory or persistent molecules that do not seem directly implicated in its main purpose. In this way, some of these constituents could be effective indicators of both local and systemic disorders even though their presence in saliva may not yet be understood [15]. However, saliva could be preferred in molecular diagnosis due to the fact that it is non-invasive and non-expensive to obtain, transport, and process the samples. The possible diagnosis value of saliva was previously emphasized, as it contains serum constituents that are transported from the local vasculature of the salivary glands into the gingival fluid flow, as Kaufman and Lamster [16] discussed. Thus, the assessment of several systemic diseases biomarkers could be performed in a more cost-effective way, and by obtaining viable biological fluid samples with less effort, training, risk, and discomfort. Moreover, it was shown that saliva sampling is preferred for a second round of collection by patients undergoing biological fluid samples collection [17]. Nevertheless, it was reported that saliva samples could provide viable sources of molecular biomarkers for delicate molecular assessments, such as DNA sequencing, as compared to blood samples [18]. Recently, Lucía Melguizo-Rodríguez [19] identified more than thirty-five salivary biomarkers descriptive for oral diseases alone, and more than 3000 species of mRNA and over 300 miRNAs, as shown in transcriptomics studies [20].

**Figure 1:**
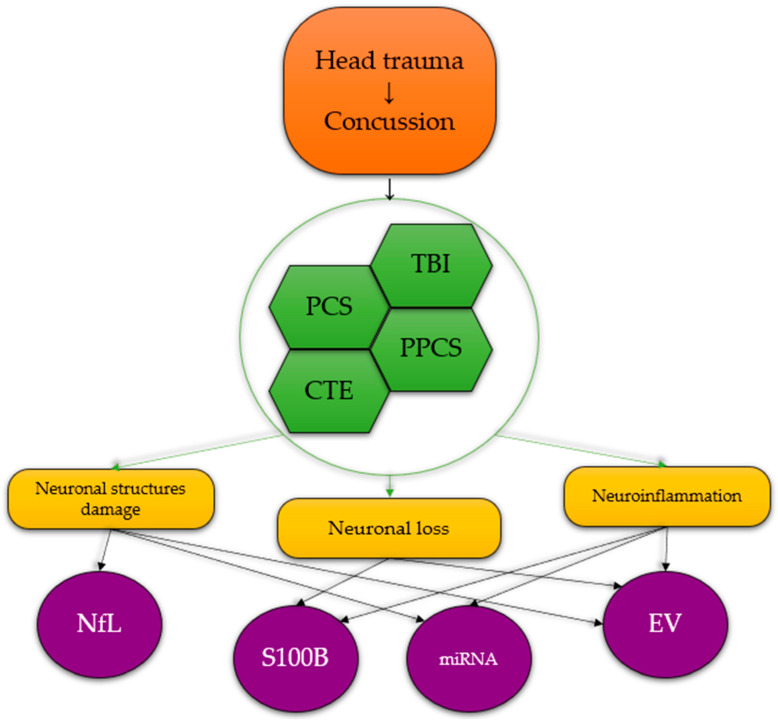
Candidate salivary biomarkers for the diagnosis of mild TBI and PCS.

As the most problematic aspect of TBIs is their diagnosis, and since the main lesions that are caused by the concussion events affect the brain tissues, the previous reports regarding the possible diagnosis biomarkers address the early identification of the possible brain damages associated with any mild head impacts that could result in concussions and TBIs [21]. In this context, recent reviews mainly described blood and CSF biomarkers, of which levels are impaired following head traumas, such as S100B protein, glial fibrillary acidic protein (GFAP), neurofilament light chain, ubiquitin C-terminal hydrolase-L1 (UCHL-1), γ-enolase, α-II spectrin, and tau protein [21,22,23]. All the mentioned molecules serve as validated biomarkers that are currently used in the classic diagnosis of brain damage. However, despite the fact that significant correlations were established between blood, CSF, and, in some cases, saliva levels, and TBIs occurrence, they are not disease-specific biomarkers, but biomarkers that indicate the effects of insults and aggressive stimuli on the brain tissues (Figure 1). The need for disease-specific biomarkers is yet stringent and unresolved for TBI, PCS, and CTE diagnosis. Since emergency care interventions are focused on fast, reliable, cost-effective, and non-invasive diagnosis, several recent reports proposed the use of saliva in concussion and TBI cases [19,21].

In this way, in TBI and PCS, a few novel studies focusing on salivary biomarkers have emphasized their importance in diagnosis [24]. As a controversy on the extracranial sources of some of these biomarkers arose, the blood–brain barrier permeability could be a limitation of blood biomarkers. However, it remains to be seen if this limitation persists for saliva biomarkers too. For this, it is important to know how molecules produced by the central nervous system end up in the saliva [25]. On this aspect, Murcko et al. [25] recently suggested that there could be many pathways by which brain-derived biomarkers distribute to the body, not necessarily by the typical way, in which tissue rupture, cellular apoptotic processes, or blood–brain barrier damage result in a cellular content influx into the circulation. In the case of TBI and PCS, previous studies showed that S100B, NfL, microRNA, and extracellular vesicles correlating with the traumatic event could be found in the saliva of the patients [19,21,24].

### 3.1. S100B

S100 proteins are a class of cytosolic proteins, involved proliferation, differentiation, migration, inflammation, apoptosis, energy metabolism, calcium balance, and protein phosphorylation [25,26], that are expressed in various tissues exerting intracellular, and/or extracellular activities. S100B is widely expressed in the central nervous system, muscles, and vascular endothelium, and is one of the members that exert both intracellular and extracellular activities. Additionally, the molecular pathways by which S100B participates could exhibit both neurotrophic and neurotoxic effects on the target tissue. Despite this, S100B mainly modulates astrocytic activation by interacting with receptors for advanced glycation end products (RAGE) of major importance in the nonenzymatic glycation and oxidation of proteins and lipids, accumulation of which determines neuroinflammation [27]. However, S100B transduction signals are also implicated in cellular responses to aggression, cellular metabolism, and gene expression [28].

Among its intracellular roles, S100B is implicated in the phosphorylation of tau protein, while it can also be secreted by the astrocytes and glial cells in a calcium modulated manner. The extracellular secreted or leaked fluid increases concentrations of S100B entering the blood stream or the cerebrospinal fluid (CSF) and promotes apoptosis [29,30]. Due to its correlation with brain damage and cellular apoptosis, S100B has been identified as a potential biomarker for TBI [31]. Despite the fact that the recent studies of Traxdorf et al. [32] described the extraneuronal origin of S100B (testis, kidneys, and adipose tissues), Murcko et a [25] showed that S100B presence in various organs does not affect its clinical significance after an episode of blood–brain barrier disruption as a result of TBI.

In a pilot study with 15 adult patients with suspected TBI and 15 control subjects, Janigro et al. [33] found that the average salivary S100B level was 3.9-fold higher than blood S100B regardless of the presence of any pathology. Similarly, they suggested that salivary S100B levels were as effective as serum levels in differentiating TBI patients from control subjects. In this way, they concluded that salivary S100B could represent an alternative to serum S100B in diagnosing TBI. Moreover, Yeung et al. [34] reported that salivary S100B could be a potent biomarker in predicting significant TBIs by evaluating 24 TBI-diagnosed children, and suggested that, although S100B is abundantly expressed in other injured/stressed tissues, such as musculoskeletal tissues, its salivary levels following TBI are significantly higher, as compared to the cases following musculoskeletal injury.

However, Monroe et al. [35] failed to demonstrate an association between S100B salivary levels and head impacts exposure while measuring the levels of S100B in the saliva of 65 water polo players before and after a competitive tournament.

### 3.2. Neurofilament Light Chain (NfL)

Neurofilament light chain (NfL) is a protein that is found throughout the central and peripheral nervous system in the axons of nerve cells under physiological conditions, and in the blood and CSF in some pathologies involving significant nerve damages [36]. NfLs are the most abundant and soluble cytosolic subunits of the neurofilaments that give structural stability to the axons, dendrites, and somas [37]. The molecular pathways through which NfL participates are mainly addressing the structural integrity of the neuronal cytoskeleton. Despite being an intracellular structural constituent of neurons, NfL could also be found in synaptic space, where they supposedly interact with NMDA GluN1 receptors by influencing their distribution [38]. Their local or systemic increased levels could indicate axonal injury and degeneration [39].

Small amounts of NfL can be released by the neurons into the interstitial space (freely communicating with the CSF and blood) in an age-dependent manner. In some pathologies (multiple sclerosis), CSF/blood NfL clinical relevance in diagnosis was compared to troponin relevance in cardiology [40].

NfL is a valuable, yet unspecific biomarker for neurodegenerative and neuronal disorders suggesting significant neuroaxonal damage. Alongside Alzheimer’s disease, Parkinson’s disease, and multiple sclerosis, both Gaetani et al. [37] and Karantali et al. [41] reviewed its efficiency to report TBI following head trauma events, when measured from blood or CSF. The latter suggested that serum NfL levels could be positively associated with the severity of TBI and the extent of PCS [41]. Due to the latency of the NfL levels peaking after TBI events, this biomarker might not be preferred in diagnostic, but rather in predicting e clinical and neuroradiological outcomes [37].

Wai et al. [42] recently explained the possible pathway of NfL molecules reaching the peripheral blood vessels by studying an experimental mouse model of malaria. Most of the excess of the unnecessary protein content within the damaged brain could be managed by the glial–lymphatic system into the nasal lymphatic vessels. However, the origin of salivary NfL is not yet fully understood, but it could be the result of pericellular capillary leak, primarily from the crevicular fluid, as Janigro et al. previously suggested [43]. There is only one study that investigated the role of salivary NfL in the diagnosis of mild TBI. Monroe et al. [35] reported that salivary NfL was directly associated with head impact frequency and cumulative head impact magnitude in their study on water polo players, concluding that repeated head impacts may cause axonal injury, even in asymptomatic athletes.

### 3.3. MicroRNAs

MicroRNAs (miRNAs) are small, non-coding RNA molecules that play essential roles in messenger RNA (mRNA) translation. MiRNAs epigenetically repress mRNA translation, based on complementary binding to their sequences, promoting mRNA degradation [44]. There are various molecular pathways in which miRNA can be detected in peripheral fluids following TBI. As more than 70% of miRNAs are expressed in the nervous system, it was shown that their functions in the neuronal tissue are closely related to local protein expression, synapse maturation, or neural circuit formation [45]. In this context, in the case of a brain traumatic event, it was shown that deregulated circulating miRNA levels were found to be related to genes belonging to molecular pathways of signal transduction in cellular response, macrophages/astrocytes activation, cellular metabolism following insults, scar formation, apoptosis, synaptic plasticity and memory formation, cellular response to cerebral ischemia and reperfusion injury, and angiogenesis [46]. Some miRNAs are currently considered potent candidates for differential diagnosis of mild and severe TBI [47].

Recently, saliva has emerged as a valuable source of miRNAs for diagnostic purposes, especially in some oral or esophageal cancers [48,49]. Saliva contains a wide range of miRNAs that are stable and easily accessible, making it a non-invasive source of biological information, in some cases regarding complete signaling pathways [44].

MicroRNAs in saliva have been studied as potential biomarkers for mild TBI and PCS. Specific miRNAs in saliva are altered in individuals with mild TBI and PCS, suggesting that these miRNAs could be used as non-invasive biomarkers for these conditions. At least nine studies have shown 188 differentially expressed miRNAs, of which 30 were detected in most cases [50]. Di Pietro et al. [51] investigated the role of salivary miRNAs on 1028 elite English rugby union players, by collecting samples during the preseason and standardized head injury assessments at in-game, post-game, and 36–48 h post-game periods. The comparison with the miRNA salivary levels in 102 uninjured and 66 players with musculoskeletal injuries showed that 32 small non-coding RNAs (snRNAs) were differently expressed across the groups. Additionally, they suggested that a combined panel of 14 sncRNAs could differentiate between concussed, non-concussed, and musculoskeletal-injured players in a window from 36 to 38 h after the traumatic event. They reported that, while at least six ncRNAs could predict PPCS with great accuracy, a panel of 11 ncRNAs combined with the age of the individuals could predict symptoms recovery [52]. In another study from the same group, saliva samples were collected from 10 concussed professional and semi-professional rugby players after 48–72 h from a mild TBI and compared to 10 non-concussed matched controls. The authors reported that 5 miRNAs were significantly upregulated in concussed athletes [52].

Fedorchak et al. [53] investigated the ability of salivary non-coding RNAs to predict PCS lasting more than 21 days and to identify recovery in cognition and balance domains. They collected 505 saliva samples from 112 individuals aged from 8 to 24 years who had sustained a mild TBI. The samples were collected within 14 days of injury and more than 21 days post-injury. They also conducted computerized balance and cognitive tests at the same time points. They reported that the individual’s age and 16 ncRNAs predicted PPCS better than the validated clinical tool, and that 11 ncRNAs showed the same accuracy for balance and cognitive test performance. They concluded that combining ncRNAs, balance, and cognition accurately identified recovery [53]. Hicks et al. [54] investigated the differences in multiple miRNAs expression in 60 children with a mild TBI, compared to 18 age-matched controls. They discovered that at least ten miRNAs demonstrated significant differences between the two groups of their study [54]. In another study from the same group, involving 13 former professional American football players with a history of recurrent concussions, and 18 age and sex-matched controls, at least 20 salivary miRNAs were found to differ between the groups, and two of them demonstrated relationships with the number of concussions [55].

Another case–control study on 455 saliva samples from 314 athletes with and without a history of a concussion showed a decrease in the expression of two miRNAs after a single episode of exercise, and an increase in 1 miRNA only after contact sports participation [56]. The same study also showed that 23 miRNAs changed at the end of a contact sports season, while two were associated with the number of head impacts sustained in a single football practice. Eleven miRNAs not confounded by exercise or season-long contact sports participation showed a significant difference between concussed and non-concussed participants, with another 6 displaying a moderate ability to identify concussions. The critical outcome of this study was that salivary miRNAs, which are not confounded by exercise, could be used as a non-invasive biomarker for assessments of concussions in athletes of contact sports [56].

Salivary miRNAs, small nucleolus RNAs, and pink-interacting RNAs have also been found helpful for the accurate diagnosis of mild TBI, in a sample of 251 individuals with mild TBI and 287 controls [57]. The authors concluded that the combination of neurocognitive testing and self-reported symptoms in the post-concussion symptom scale, with these non-coding RNAs, are superior to clinical testing or salivary biomarkers alone.

Salivary miRNAs expression is also a sensitive marker and has prognostic value for prolonged post-concussion symptoms [58] and can differentiate acute concussion syndrome from PCS. Different miRNAs are associated with post-concussion symptoms, such as headaches, memory difficulties, and fatigue.

A subset of salivary and serum miRNAs predicted the likelihood of a TBI and demonstrated quantitative associations with head impacts and cognitive and balance measures in a group of twenty-four martial arts fighters, and salivary miRNAs demonstrated far more utility [59]. Miller et al. [60] identified thirteen salivary miRNAs with significantly various levels in children with a history of mild TBI and with or without PPCS.

### 3.4. Extracellular Vesicles

The role of extracellular vesicles (EVs) as a mechanism of cell-to-cell communication has been widely described in the literature [61]. These vesicles are released by cells, including stem cells and progenitors, and interact with target cells by transferring surface receptors, proteins, mRNA, and bioactive lipids through surface-expressed ligands. In this way, the plethora of molecular pathways that implicate the formation of extracellular vesicles are usually based on signal transduction modulation and cellular communication. In this way, exosomes, microvesicles, and apoptotic bodies are potent means of protein dissemination during TBI progression [62].

Moreover, EVs were shown to participate in the removal of unwanted active molecules (for example, in AD, EVs are thought to be one of the milieus through which the pathogenic proteins are spread) [63,64]. The possible roles of EVs in TBI were recently described by Khan et al. [62], who mainly highlighted the clinical implications in the therapeutic perspectives of EVs. However, despite the fact that the difficulties in isolating EVs are surpassed by novel techniques [65,66,67], EVs’ implications in TBI remain a controverted and unsolved puzzle. The main way they end up in the peripheral fluids, including sweat and saliva, is closely tied to their excellent ability to cross the blood–brain CNS barrier [68]. Most of the recent studies on EVs in TBI are based on the evaluation of EVs isolated from blood or CSF, and only a few were concentrated on salivary EVs.

As a matter of fact, one of the most extensive study on salivary EVs in TBI, based on 54 subjects (23 controls with no history of head traumas, 16 patients from an outpatient concussion clinic, and 15 patients from the emergency department who had sustained a head trauma within 24 h), showed that salivary EVs’ genes expression could serve as a viable source of biomarkers for mild TBI, and also described the possible genetic overlap between AD and TBI, in terms of similarities of brain injury mechanisms [69]. A smaller yet relevant study, based on 8 mixed martial arts fighters and 7 controls, showed that salivary EVs could be potential biomarkers of the acute phase of brain trauma in correlation with injury severity [70]. Moreover, Cheng et al. [71], in a study based on 6 TBI patients, 6 concussion patients, and 7 healthy controls, identified a panel of multiple genes whose expression was altered following brain trauma events: 9 upregulated genes in acute TBI, and 13 upregulated genes in concussion (Table 2).

Despite these promising results, the evidence regarding the assessment of salivary biomarkers in TBI, as well as their dynamics and purpose in diagnosis and/or prognosis, remain scarce. In this way, for further studies that would emphasize the correlation between the cranial origin of TBI biomarkers and their extracranial destination, focusing on salivary sources could shed more light on these aspects. Nevertheless, a clearer representation of saliva biomarker dynamics could provide significant background knowledge, meaning they could be classified in diagnosis and/or prognosis biomarkers based on the presence, timing, and peak levels in the saliva samples of the patients that have undergone TBI events.

## 4. Conclusions

Mild TBIs are a major public health issue, affecting more than 69 million patients a year worldwide. PCS, although widely recognizable, remains a controversial entity. Both mild TBI and PCS are currently clinically diagnosed based on symptomatology and, in some cases, brain imaging evaluation. The current state of research on saliva biomarkers and their clinical applications is promising, but most studies focused on miRNAs, and only a few studies investigated the role of EVs, NfL, and S100B. The critical outcome of salivary biomarkers’ current state is that miRNAs and other non-coding RNAs, combined with clinical history and examination, self-reported symptoms, and clinical–paraclinical cognitive and balance testing, can provide a non-invasive alternative diagnostic methodology to the currently approved plasma and cerebrospinal fluid biomarkers.

Despite these promising results, more research is required before salivary biomarkers can be widely adopted in clinical practice. In particular, extensive, well-designed clinical studies are needed to validate saliva biomarkers’ accuracy and reliability, and determine the optimal methods for measuring and interpreting these biomarkers. Thus, the current state of research on saliva biomarkers and their clinical applications is promising, but more research is needed to fully understand the role of these biomarkers in disease diagnosis and management.

## Figures and Tables

**Table 1:** Mayo system of TBIs classification [8,9].

	Criteria
Possible	Neurocognitive symptoms: blurred vision, confusion, headache, or nausea.
Probable—mild	Neurocognitive symptoms: loss of consciousness (<30 min), post-traumatic amnesia (<24 h); Mechanical damage: depressed, basilar, or linear skull fracture; Brain damage: intact *dura matter*.
Definite moderate—severe	Neurocognitive symptoms: loss of consciousness (>30 min), post-traumatic amnesia (>24 h); GCS < 13; Brain damage: brain hematoma, brain hemorrhage, contusions, or ruptured dura mater. Death.

**Table 2:** Studies that evaluated salivary biomarkers in head trauma and concussion.

Study	Experimental Design	Results
S100B		
[33]	15 adult patients with suspected TBI and 15 control subjects	Average salivary S100B level 3.9-fold higher than blood S100B. Salivary S100B levels are as effective as serum levels in differentiating TBI patients from control subjects.
[34]	70 children: 24 acute and isolated TBI 46 with musculoskeletal injuries only	Salivary S100B levels following TBI are significantly higher, as compared to the ones following musculoskeletal injury
[35]	65 water polo players, before and after a competitive tournament	No association between S100B salivary levels and head impacts exposure.
Neurofilaments light chain (NfL)		
[35]	65 water polo players, before and after a competitive tournament	Salivary NfL is directly associated with head impact frequency and cumulative head impact magnitude, as compared with baseline salivary NfL.
Micro RNAs (miRNAs)		
[49]	1028 rugby players, 66 players with musculoskeletal injuries, 102 uninjured players	Significant difference sin expression of 32 small non-coding RNAs: 14 small non-coding RNAs—concussed players versus players without injuries 6 small non-coding RNAs—prolonged PPCS prediction 11 small non-coding RNAs—age dependent symptoms recovery prediction
[52]	10 concussed professional and semi-professional rugby players 10 non-concussed matched controls	5 miRNAs significantly upregulated in concussed athletes
[53]	112 mild TBI individuals (8 to 24 years old)	16 non-coding RNAs—PPCS prediction
[54]	60 children diagnosed with mild TBI 18 age-matched controls	10 miRNAs expression significantly altered in mild TBI children
[55]	13 former professional American football players, 18 age and sex-matched controls	20 salivary miRNAs expression significantly altered in athletes 2 salivary miRNAs associated with the number of concussion events
[56]	314 athletes with and without a history of a concussion	2 miRNAs ↓ after physical exercise 1 miRNA ↑ after contact sports participation 23 miRNAs expression altered after 1 season of contact sports 2 miRNAs associated with head impacts number Significant differences in 11 miRNAs expression in concussed versus non-concussed participants
Extracellular vesicles (EVs)		
[69]	54 subjects: 16 post-concussion patients, 15 head trauma patients, 23 controls	Salivary EVs gene expression—viable source of biomarkers for mild TBI Multiple Alzheimer’s disease genes expression present in post-mild TBI saliva samples:
[70]	8 mixed martial arts fighters, 7 from controls.	EVs could be potential biomarkers of the acute phase of brain trauma in correlation with injury severity.
[71]	6 TBI patients, 6 concussion patients, 7 healthy controls	9 upregulated genes in acute TBI: LOX5, ANXA3, CASP1, IL2RG, ITGAM, ITGB2, LTA4H, MAPK14, and TNFRSF1A, 13 upregulated genes in concussion: ADRB1, ADRB2, BDKRB1, HRH1, HRH2, LTB4R2, LTB4R, PTAFR, CYSLTR1, CES1, KLK1, MC2R, and PTGER3.
